# Supplementary material for: Adenosine receptor 2a agonists target mouse CD11c+T-bet+ B cells in infection and autoimmunity
Source: Nat Commun. 2022 Jan 21;13:452. doi: 10.1038/s41467-022-28086-1 (PMC8782827; doi:10.1038/s41467-022-28086-1)
Supplement: Supplementary file 1 — Supplementary Information [file 41467_2022_28086_MOESM1_ESM.pdf]

Supplementary Figure 1

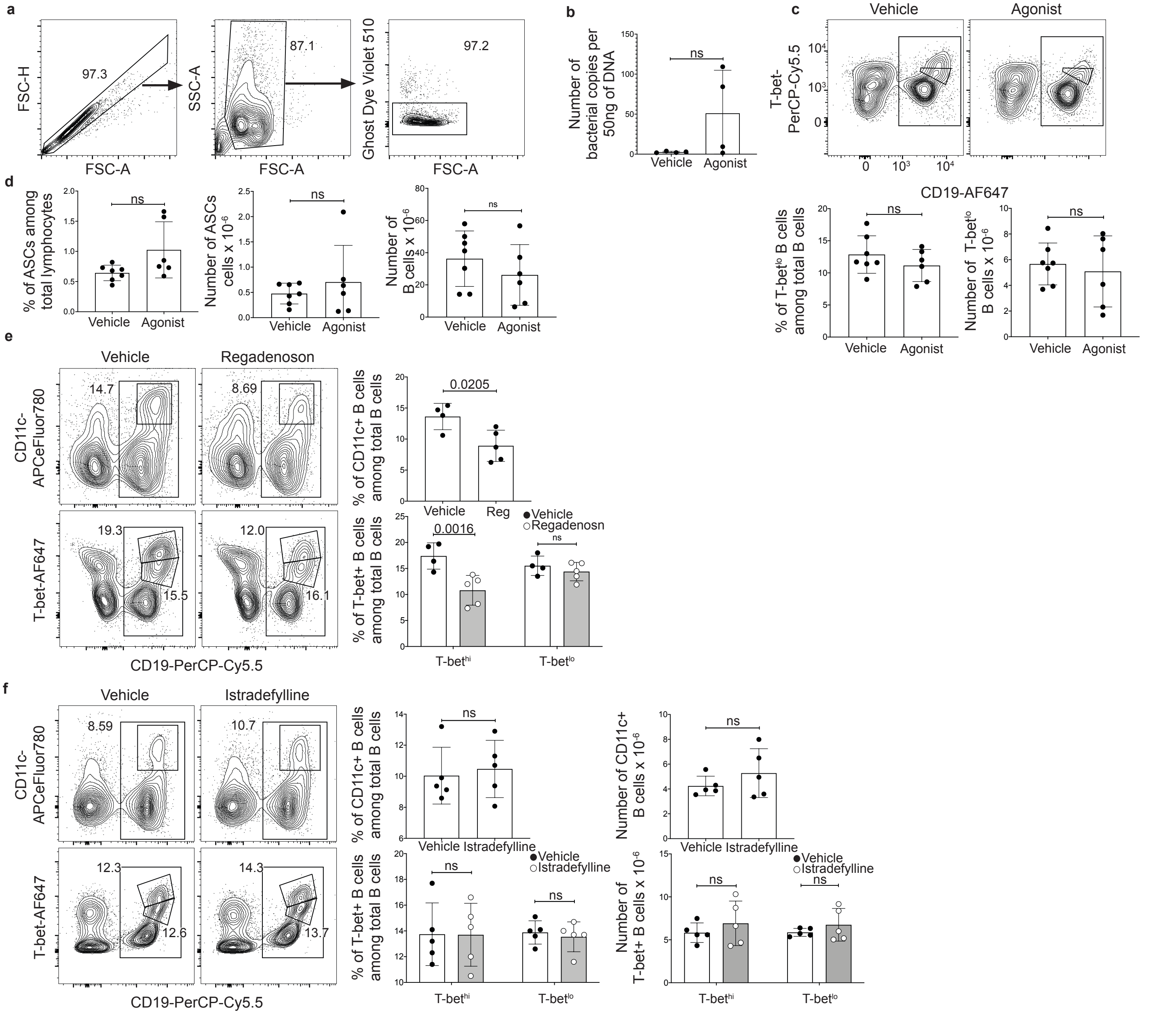

Supplementary Figure 1: A_{2A} receptor agonists, but not antagonists, target CD11c+ T-bet+ B cells
(A) Cells analyzed by flow cytometry were pre-gated on singlets and lymphocytes, as shown in the contour plots. Cells from figures 2a-b, and 4a-b were also gated on live cells as shown in the contour plots.
(B) Livers from vehicle or CGS-21680-treated mice from figure 1A were analyzed for bacterial load by qPCR (n = 4). The graph shows the number of bacterial copies for each mouse. Statistical significance was determined using two-tailed un-paired t tests.
(C) Splenocytes from the mice in figure 1A were analyzed by flow cytometry (vehicle; n = 7, agonist; n = 6). The contour plots and graphs show the percentages and numbers of T-bet^{lo} CD19+ cells. Graphs represent aggregate data from two independent experiments. Statistical significance was determined using two-tailed un-paired t tests.
(D) Splenocytes from the mice in figure 1A were analyzed by flow cytometry (vehicle; n = 7, agonist; n = 6). The graphs show the percentages and numbers of total B cells and CD138+ cells. Graphs represent aggregate data from two independent experiments. Statistical significance was determined using two-tailed un-paired t tests.
(E) *E. muris*-infected female C57BL6/J mice were treated with vehicle (n = 4) or Regadenoson (n = 5) every other day for seven days starting on day 30 post-infection; splenocytes analyzed on day 37 post-infection. The contour plots and graphs show the percentages of CD11c+ CD19+, T-bet^{hi} CD19+, and T-bet^{lo} CD19+ cells. Statistical significance was determined using a two tailed un-paired t test (top) and an ordinary two-way ANOVA with Sidak' multiple comparisons test (bottom, df = 14).
(F) *E. muris*-infected female C57BL6/J mice were treated with vehicle (n = 5) or Istradefylline (n = 5) every other day for seven days starting on day 30 post-infection; splenocytes were analyzed on day 37 post-infection. The contour plots and graphs show the percentages and numbers of CD11c+ CD19+, T-bet^{hi} CD19+, and T-bet^{lo} CD19+ cells. Statistical significance was determined using two tailed un-paired t tests (top) and ordinary two-way ANOVAs with Sidak' multiple comparisons test (bottom, df = 16). Columns and error bars indicate the arithmetic mean and SD.

Supplementary Figure 2

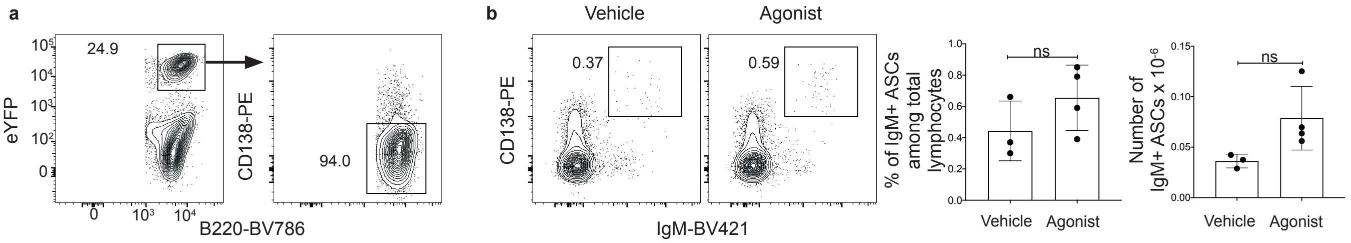

Supplementary Figure 2: eYFP labeled T-bet+ B cells are depleted by A_{2A} receptor stimulation

(A) Splenocytes from the mice in figure 1C were analyzed by flow cytometry. The dot plots show the percentage of CD138-negative cells among eYFP+ B cells. Columns and error bars indicate the arithmetic mean and SD.

(B) Bone marrow from the mice in figure 1C was analyzed by flow cytometry (vehicle; n = 3, agonist; n = 4). The contour plots and graphs show the percentages and numbers of CD138+ IgM+ cells. Statistical significance was determined using two-tailed unpaired t tests. Columns and error bars indicate the arithmetic mean and SD.

Supplementary Figure 3

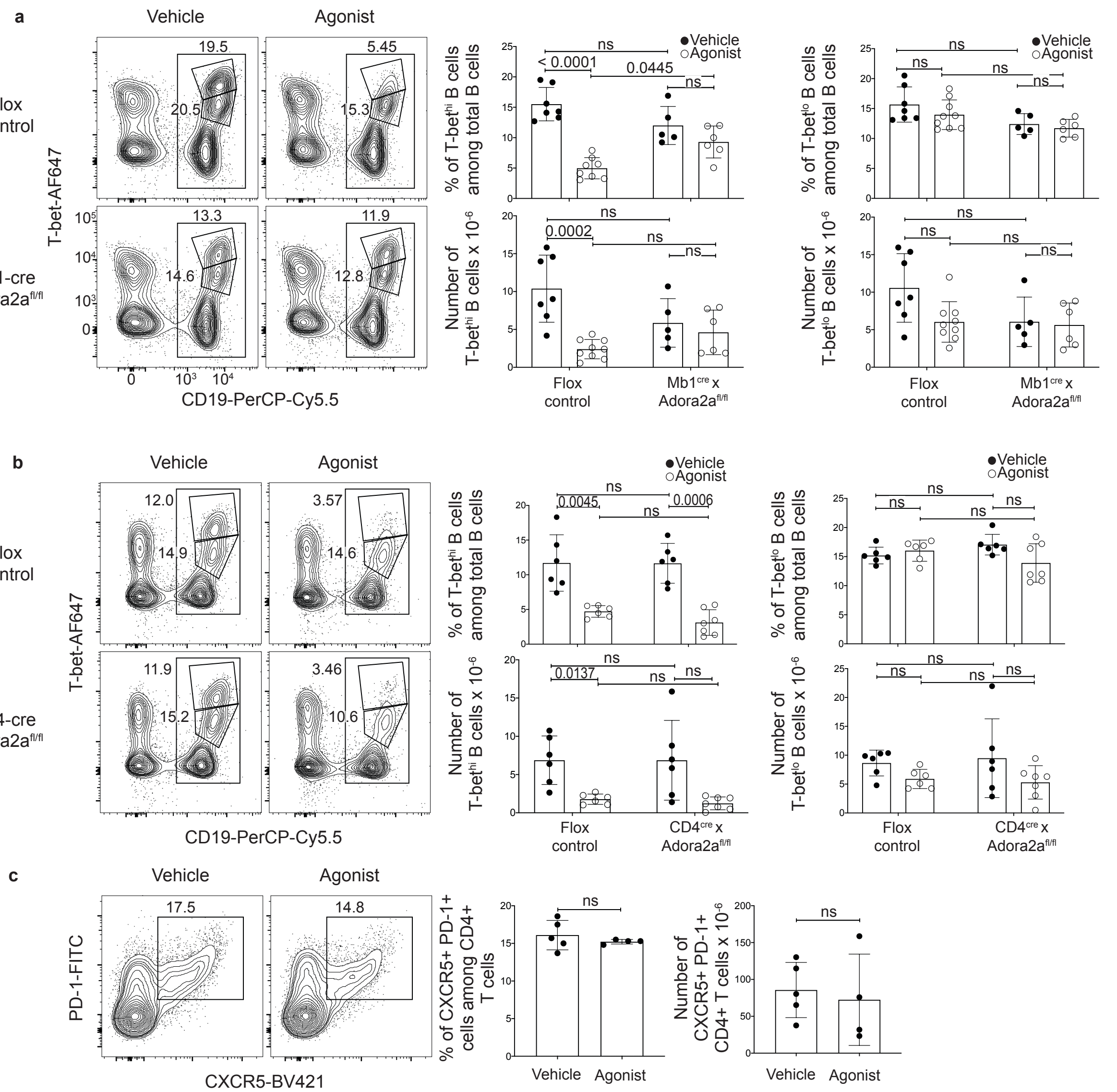

Supplementary Figure 3: A_{2A} receptor agonists directly target CD11c+ T-bet+ B cells

(A) Splenocytes from the mice in figure 1D were analyzed by flow cytometry (vehicle control: n = 7; vehicle Mb1cre/+ x Adora2aflox/flox: n = 6; agonist control: n = 9; agonist Mb1cre/+ x Adora2aflox/flox: n = 7). The contour plots and graphs show the percentages and numbers of T-bet^{hi} CD19+ and T-bet^{lo} CD19+ cells. Statistical significance was determined using two-way ANOVAs with Sidak's multiple comparisons test (df = 23). Columns and error bars indicate the arithmetic mean and SD.

(B) Splenocytes from the mice in figure 1E were analyzed by flow cytometry (vehicle control: n = 6; vehicle CD4cre/+ x Adora2aflox/flox: n = 6; agonist n = 7) . The contour plots and graphs show the percentages and numbers of T-bet^{hi} CD19+ and T-bet^{lo} CD19+ cells. Statistical significance was determined using two-way ANOVAs with Sidak's multiple comparisons test(df = 19). Columns and error bars indicate the arithmetic mean and SD.

(C) Splenocytes from the mice in figure 1A were analyzed by flow cytometry (vehicle; n = 7, agonist; n = 6). The contour plots and graphs show the percentages and numbers of PD-1+ CXCR5+ cells among CD3+ CD4+ T cells. Statistical significance was determined using two-tailed un-paired t tests. Columns and error bars indicate the arithmetic mean and SD.

Supplementary Figure 4

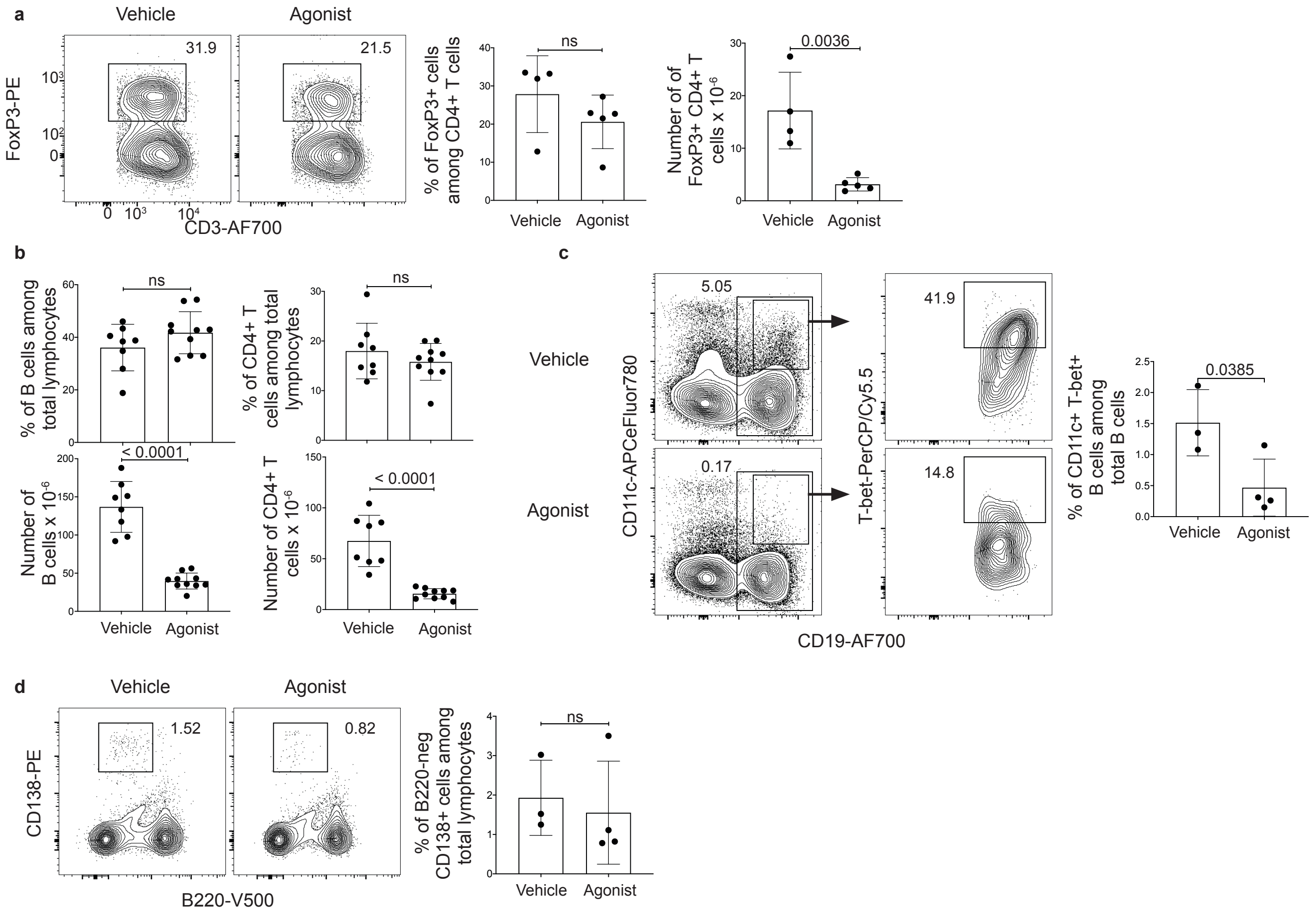

Supplementary Figure 4: A_{2A} receptor agonists target T-bet+ B cells in multiple mouse models of lupus

(A) Splenocytes from the mice in figure 2A were analyzed by flow cytometry (vehicle: n = 8; agonst: n = 10). The contour plots and graphs show the percentages and numbers of FoxP3+ T cells among CD4+ CD3+ T cells. Statistical significance was determined using two-tailed un-paired t tests and a Mann-Whitney test (left graph). Columns and error bars indicate the arithmetic mean and SD.

(B) Splenocytes from the mice in figure 2A were analyzed by flow cytometry (vehicle: n = 8; agonst: n = 10). The graphs show the percentages of B cells (p=0.1744) and CD4+ T cells (0.3355) among total lymphocytes and the number of B cells (p<0.0001) and CD4+ T cells (p<0.0001). Statistical significance was determined using two-tailed un-paired t tests. Columns and error bars indicate the arithmetic mean and SD.

(C) Nine-month-old SLE1.2.3. mice were treated with vehicle (n = 3) or agonist (n = 4) every other day for 5 days and splenocytes analyzed. The zebra plots and graphs show the percentages of CD19+ CD11c+ T-bet+ B cells. Statistical significance was determined using a two-tailed un-paired t test. Columns and error bars indicate the arithmetic mean and SD.

(D) Splenocytes from the mice in Supplementary figure S4C were analyzed by flow cytometry (vehicle: n = 3; agonist: n = 4). The contour plots and the graph show the percentages of CD138+ B220-negative cells. Statistical significance was determined using a two-tailed Mann-Whitney test. Columns and error bars indicate the arithmetic mean and SD.

Supplementary Figure 5

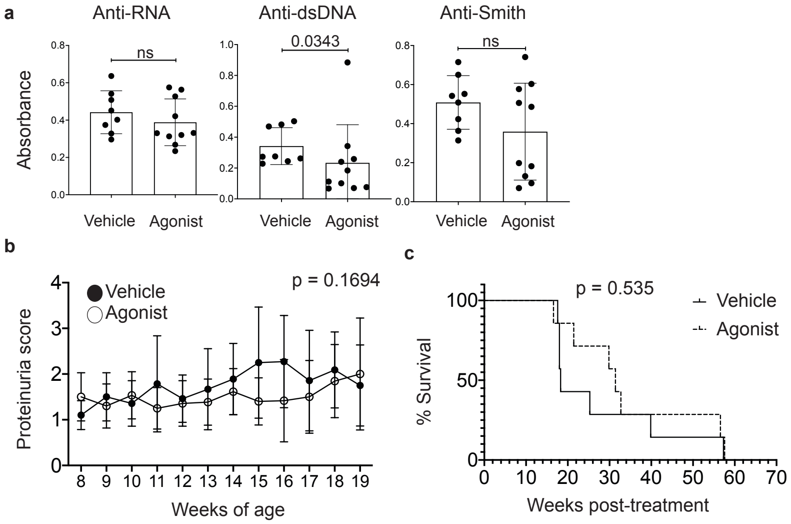

Supplementary Figure 5: A_{2A} receptor agonism ameliorated disease in a mouse model of lupus
(A) Sera from the mice in figure 2A were analyzed for IgG2a anti-RNA, anti-dsDNA, and anti-Smith antibodies by ELISA (vehicle: n = 8; agonst: n = 10). The graphs show relative absorbance values for the analyzed sera. Statistical significance was determined using a two-tailed un-paired t test or a Mann-Whitney test (middle plot). Columns and error bars indicate the arithmetic mean and SD.
(B) Urine samples from the mice in figure 2A were analyzed for albumin (vehicle: n = 8; agonst: n = 10). The graph shows the proteinuria scores from analyzed urine. Statistical significance was determined using a mixed-effect model with Sidak's multiple comparisons test. Differences were not statistically significant (p = 0.1694). Columns and error bars indicate the arithmetic mean and SD.
(C) Eight-week-old female MRL/lpr mice were treated with vehicle (n = 7) or agonist (n = 8) twice weekly and sacrificed when moribund. Median survival times for the vehicle and agonist-treated mice were 32.1 weeks (95% CI: 16.6-57.6) and 21.8 weeks (95% CI: 17.6-57.3). The observed difference was not statistically significant (p = 0.535). Statistical significance between median survival times was determined by inverting the Kaplan-Meier survival curve and standard error was estimated by bootstrap. Columns and error bars indicate the arithmetic mean and SD.

Supplementary Figure 6

a

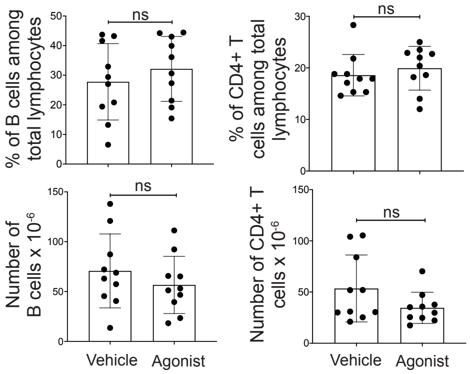

Supplementary Figure 6: A_{2A} receptor stimulation did not affect the total number of B cells or CD4+ T cells (A) Splenocytes from the mice in figure 4A were analyzed by flow cytometry (n = 10). The graphs show the percentages of B cells and CD4+ T cells among total lymphocytes and the number of B cells, CD4+ T cells, and splenocytes. Statistical significance was determined using a two-tailed un-paired t test or a Mann-Whitney test. Columns and error bars indicate the arithmetic mean and SD.
