# Supplementary material for: Adenosine receptor 2a agonists target mouse CD11c+T-bet+ B cells in infection and autoimmunity
Source: Nat Commun. 2022 Jan 21;13:452. doi: 10.1038/s41467-022-28086-1 (PMC8782827; doi:10.1038/s41467-022-28086-1)
Supplement: Supplementary file 3 — Reporting Summary [file 41467_2022_28086_MOESM3_ESM.pdf]

nature portfolio

Corresponding author(s): Gary Winslow

Last updated by author(s): October 16, 2021

Reporting Summary

Nature Portfolio wishes to improve the reproducibility of the work that we publish. This form provides structure for consistency and transparency in reporting. For further information on Nature Portfolio policies, see our Editorial Policies and the Editorial Policy Checklist.

Statistics

For all statistical analyses, confirm that the following items are present in the figure legend, table legend, main text, or Methods section.

n/a	Confirmed	
☐	☒	The exact sample size (n) for each experimental group/condition, given as a discrete number and unit of measurement
☐	☒	A statement on whether measurements were taken from distinct samples or whether the same sample was measured repeatedly
☐	☒	The statistical test(s) used AND whether they are one- or two-sided<br>*Only common tests should be described solely by name; describe more complex techniques in the Methods section.*
☐	☒	A description of all covariates tested
☐	☒	A description of any assumptions or corrections, such as tests of normality and adjustment for multiple comparisons
☐	☒	A full description of the statistical parameters including central tendency (e.g. means) or other basic estimates (e.g. regression coefficient) AND variation (e.g. standard deviation) or associated estimates of uncertainty (e.g. confidence intervals)
☐	☒	For null hypothesis testing, the test statistic (e.g. F, t, r) with confidence intervals, effect sizes, degrees of freedom and P value noted<br>*Give P values as exact values whenever suitable.*
☒	☐	For Bayesian analysis, information on the choice of priors and Markov chain Monte Carlo settings
☒	☐	For hierarchical and complex designs, identification of the appropriate level for tests and full reporting of outcomes
☐	☒	Estimates of effect sizes (e.g. Cohen's d, Pearson's r), indicating how they were calculated

Our web collection on statistics for biologists contains articles on many of the points above.

Software and code

Policy information about availability of computer code

Data collection	Flow cytometry data were acquired on a BD Fortessa flow cytometer using Diva software (BD Bioscience)
Data analysis	Data were analyzed with FlowJo software version 10.7 (BD Bioscience) and statistical analysis was performed using Prism 9 (GraphPad).

For manuscripts utilizing custom algorithms or software that are central to the research but not yet described in published literature, software must be made available to editors and reviewers. We strongly encourage code deposition in a community repository (e.g. GitHub). See the Nature Portfolio guidelines for submitting code & software for further information.

Data

Policy information about availability of data

All manuscripts must include a data availability statement. This statement should provide the following information, where applicable:

- Accession codes, unique identifiers, or web links for publicly available datasets
- A description of any restrictions on data availability
- For clinical datasets or third party data, please ensure that the statement adheres to our policy

Data generated from this manuscript are available upon request.

Field-specific reporting

Please select the one below that is the best fit for your research. If you are not sure, read the appropriate sections before making your selection.

☒ Life sciences ☐ Behavioural & social sciences ☐ Ecological, evolutionary & environmental sciences

For a reference copy of the document with all sections, see nature.com/documents/nr-reporting-summary-flat.pdf

Life sciences study design

All studies must disclose on these points even when the disclosure is negative.

Sample size	Sample size was calculated based on previous data (e.g., Levack et al, The Journal of Immunology, 2020) assuming a power of 80% and an alpha value of 0.05.
Data exclusions	Two mice were excluded from Figure 1c as they had been misgenotyped.
Replication	Replicates are noted in figure legends. Replicate data reproduced the initial observations.
Randomization	Littermate mice were randomly assigned to control or experimental groups following genotyping.
Blinding	Blinding was not possible as the same scientist performed the treatment and analysis. However, blinding was not necessary as mice were randomly assigned to treatment groups prior to treatment.

Reporting for specific materials, systems and methods

We require information from authors about some types of materials, experimental systems and methods used in many studies. Here, indicate whether each material, system or method listed is relevant to your study. If you are not sure if a list item applies to your research, read the appropriate section before selecting a response.

Materials & experimental systems

n/a	Involved in the study	
☐	☒	Antibodies
☒	☐	Eukaryotic cell lines
☒	☐	Palaeontology and archaeology
☐	☒	Animals and other organisms
☒	☐	Human research participants
☒	☐	Clinical data
☒	☐	Dual use research of concern

Methods

n/a	Involved in the study	
☒	☐	ChIP-seq
☐	☒	Flow cytometry
☒	☐	MRI-based neuroimaging

Antibodies

Antibodies used

anti-CD16/32 (clone 2.4G2, produced in house and used undiluted in media from source hybridoma)
PerCpCy5.5-conjugated anti-CD19 (clone 6D5, BioLegend, cat# 115533, dilution 1:200)
Alexafluor 700-conjugated anti-CD19 (clone 6D5, BioLegend cat# 115528, dilution 1:200)
APC-eFluor 780-conjugated anti-CD11c (clone N418, eBioscience cat# 47-0114-82, dilution 1:200)
Brilliant Violet 785-conjugated anti-B220 (clone RA3-6B2, BioLegend cat# 103245, dilution 1:200)
V500-conjugated anti-B220 (clone RA3-6B2, BD Horizon cat# 561227, dilution 1:100)
Alexafluor 647-conjugated anti-T-bet (clone 4B10, BioLegend cat# 644803, dilution 1:400)
PerCpCy5.5-conjugated anti-T-bet (clone 4B10, BioLegend cat# 644805, dilution 1:400)
FITC-conjugated anti-PD-1 (clone 29F.1A12, BioLegend cat# 135214, dilution 1:100)
PerCpCy5.5-conjugated anti-CD4 (clone RM4-4, BioLegend cat# 116011, dilution 1:200)
Alexafluor 700-conjugated anti-CD3 (clone 17A2, BD Pharmigen cat# 561388, dilution 1:400)
Brilliant Violet 421-conjugated anti-CXCR5 (clone L138D7, BioLegend cat# 145511, dilution 1:200)
PE-conjugated anti-CD138 (clone 281-2, BioLegend cat# 142504, dilution 1:200)
Brilliant Violet 421-conjugated anti-IgM (clone RMM-1, BioLegend cat# 406517, dilution 1:200)
PE-conjugated anti-FoxP3 (clone 150D, BioLegend cat# 320007, dilution 1:200)
Alkaline Phosphatase-conjugated anti IgG2 (clone polyclonal, SouthernBiotech, cat# 0107-04, dilution 1:1000)
Alkaline Phosphatase-conjugated anti IgG2a (clone polyclonal, SouthernBiotech, cat# HOPC-1, dilution 1:1000)

Validation

Antibodies were validated by their respective manufacturers using either surface or intracellular immunofluorescent staining on mouse cells.

Animals and other organisms

Policy information about studies involving animals; ARRIVE guidelines recommended for reporting animal research

Laboratory animals	C57BL/6J, CD4cre (B6.Cg-Tg(Cd4-cre)1Cwi/BfluJ), Mb1cre (B6.C(Cg)-Cd79atm1(cre)Reth/EhobJ), MRL/lpr (MRL/MpJ-Faslpr/J), SLE123 (B6;NZM-Sle1NZM2410/Aeg Sle2NZM2410/Aeg Sle3NZM2410/Aeg/LmoJ), and Rosa26eYFP (B6.Cg-Gt(ROSA)26Sortm3(CAG-EYFP)Hze/J) mice were obtained from The Jackson Laboratory (Bar Harbor, ME). T-bet-creERT2 mice were generated by Dr. Lin Gan at the University of Rochester, Rochester, NY. Adora2aflox (B6;129-Adora2atm1Dyj/J) mice were provided by Dr. Joel Linden, La Jolla Institute for Immunology, La Jolla, CA. All mice were housed and bred in the SUNY Upstate Medical University Animal Care Facility (Syracuse, NY), in accordance with institutional guidelines for animal welfare. Mice were housed on a 12 hour light/dark cycle at a temperature of approximately 23C and humidity of approximately 35%. All mice used for experiments were at least 6 weeks old, and both male and female mice were used unless otherwise stated.
Wild animals	No wild animals were used in the present study.
Field-collected samples	No field collect samples were used in the present study.
Ethics oversight	All studies involving animals were approved by the SUNY Upstate Medical University Institutional Animal Care and Use Committee NYSDOH Unit A073.

Note that full information on the approval of the study protocol must also be provided in the manuscript.

Flow Cytometry

Plots

Confirm that:

☒ The axis labels state the marker and fluorochrome used (e.g. CD4-FITC).

☒ The axis scales are clearly visible. Include numbers along axes only for bottom left plot of group (a 'group' is an analysis of identical markers).

☒ All plots are contour plots with outliers or pseudocolor plots.

☒ A numerical value for number of cells or percentage (with statistics) is provided.

Methodology

Sample preparation	Spleens and lymph nodes were disaggregated using a 70um cell strainer (BD Falcon). Erythrocytes were removed by incubation with ACK lysis Buffer (Quality Biological Inc). Cells were treated with anti-CD16/32 (2.4G2) and mouse cells were incubated with surface antibodies.<br><br>Livers were perfused with PBS and disaggregated using a 70um cell strainer (BD Falcon). Cells were gradient separated using 40% Percoll (Sigma Aldrich) and erythrocytes were removed by incubation with ACK lysis Buffer (Quality Biological Inc).<br><br>The cells were stained at 4C for 30 min, washed, and analyzed. For intracellular staining, surface-stained cells were fixed/permeabilized for 40 minutes at 4C, using the Transcription Factor Buffer set Fixation/permeabilization buffer (BD Pharmingen), washed, stained at 4C for 30 minutes, washed, and analyzed. Unstained cells were used to establish the flow cytometer voltage settings, and single-color positive controls were used to adjust compensation.
Instrument	BD Fortessa flow cytometer
Software	FlowJo version 10.7 and Diva software (BD Bioscience)
Cell population abundance	No cell sorting was performed in the present study.
Gating strategy	All cells analyzed by flow cytometry were pre-gated diagonally on singlets based on FSC-H vs FSC-A. Cells were gated on lymphocytes based on FSC-A and SSC-A.

☒ Tick this box to confirm that a figure exemplifying the gating strategy is provided in the Supplementary Information.
